# Randomised open label exploratory, safety and tolerability study with calmangafodipir in patients treated with the 12-h regimen of N-acetylcysteine for paracetamol overdose—the PP100–01 for Overdose of Paracetamol (POP) trial: study protocol for a randomised controlled trial

**DOI:** 10.1186/s13063-018-3134-1

**Published:** 2019-01-08

**Authors:** The POP Trial Investigators, Ruaridh Buchan, Thomas Caparrotta, Michael Eddleston, Emma E. Morrison, Matthew Reed, Robert J. Lee, Garry Milne, Lynsey Milne, Katherine Oatey, Phillip Rayson, Michelle Steven, Christopher J. Weir, Polly Black, Caroline Blackstock, Rachel O’Brien, Bernadette Gallagher, Julia Grahamslaw, Adam Lloyd, Allan Macraild, Megan McGrath, Mary Morrissey, Emma Ward, Liz Hasseld, Mia Paderanga, Miranda Odam, Dennis Henriksen, Marie Bengtson, James W. Dear, Wilna Oosthuyzen, James Dear

**Affiliations:** The Queen’s Medical Research Institute, University of Edinburgh, 47 Little France Crescent, Edinburgh, EH16 4TJ UK

**Keywords:** Paracetamol, Acetylcysteine, Overdose, Hepatotoxicity, Calmangafodipir

## Abstract

**Background:**

Paracetamol (acetaminophen) overdose (POD) is the commonest cause of acute liver failure in Europe and North America. Current treatment involves the use of the antidote N-acetylcysteine (NAC) in patients deemed at risk of liver damage. This regimen was introduced in the 1970s and has remained largely unchanged even though the initial NAC infusion is frequently associated with adverse reactions, in particular nausea, vomiting, and anaphylactoid reactions. NAC has reduced efficacy for preventing liver injury in those patients who present later after overdose. We designed a randomised study investigating the safety and tolerability of a superoxide dismutase (SOD) mimetic, calmangafodipir (PP100–01), co-treatment with a 12-h NAC regimen compared with NAC treatment alone in patients with POD.

**Methods/design:**

We have designed an open-label, randomised, exploratory, rising dose design, NAC-controlled, phase 1 safety and tolerability study in patients treated with NAC for POD. A total of 24 patients will be assigned into one of three dosing cohorts of eight patients (*n* = 6 for PP100–01 and NAC; *n* = 2 for NAC alone). The doses of PP100–01 are 2, 5, and 10 μmol/kg. The primary outcome is the safety and tolerability of PP100–01 when co-administered with a 12-h NAC regimen compared with NAC treatment alone. Furthermore, the study will explore if PP100–01 has potential efficacy for the treatment of paracetamol-induced liver injury by measurement of conventional clinical and exploratory biomarkers.

**Discussion:**

The aim of the study is to test the safety and tolerability of a SOD mimetic, PP100–01, in combination with a 12-h NAC regimen in patients presenting within 24 h of POD. This study will provide valuable data regarding the incidence of adverse events caused by the 12-h NAC plus PP100–01 regimen and may provide evidence of PP100–01 efficacy in the treatment of paracetamol-induced liver injury.

**Trial registration:**

EudraCT, 2017–000246-21; ClinicalTrials.gov, NCT03177395. Registered on 6 June 2017.

**Electronic supplementary material:**

The online version of this article (10.1186/s13063-018-3134-1) contains supplementary material, which is available to authorized users.

## Background

Paracetamol/acetaminophen (N-acetyl-p-aminophenol (APAP)) is the most common agent taken as an overdose in the UK. APAP is ingested by approximately 40% of patients admitted to hospital with self-harm. Annually, overdose directly leads to around 100,000 hospital attendances in the UK with around half of these patients being admitted to hospital for emergency antidote treatment [1]. APAP is responsible for the deaths of around 100–150 people per year in the UK [2]. In the USA, APAP overdose accounts for more than 56,000 hospital attendances and around 450 deaths due to acute liver failure each year [3].

In paracetamol overdose (POD), the normal APAP detoxification pathways (sulphation and glucuronidation) are overwhelmed, leading to the formation of the reactive intermediate metabolite, N-acetyl-p-benzoquinoneimine (NAPQI), which binds covalently to liver proteins resulting in cell death. Experimental animal data demonstrate that APAP-induced toxicity occurs in two phases: an initial metabolic phase followed by an oxidative phase. In human patients, the metabolic phase is predominantly during the first 8 h (0–8 h) with the oxidative phase dominating thereafter (> 8 h). During the metabolic phase, APAP metabolites are conjugated and excreted via the kidneys. In the oxidative phase the glutathione (GSH) stores are depleted and the reactive metabolite NAPQI binds to liver proteins with increased oxidative stress and, ultimately, loss of mitochondrial membrane potential and subsequent cell death [4]. Oxidative stress can directly trigger mitochondrial membrane permeability with pore opening and collapse of the mitochondrial membrane potential. The toxicity of reactive oxygen and reactive nitrogen species is potentiated by the fact that mitochondrial GSH levels are severely depleted during the APAP metabolism, which leaves these organelles highly vulnerable to injury.

In humans, APAP-induced liver injury is usually asymptomatic in its early stages. Nausea, vomiting, and abdominal pain are common soon after overdose but are not specific for liver injury. As toxicity progresses, the patient may experience pain in the region of the liver. In severe poisoning, hepatic encephalopathy and acute kidney injury can occur and these features are used to identify patients that need liver transplantation to avoid death. Acetylcysteine (N-acetylcysteine (NAC)) was developed as an antidote for APAP poisoning in the 1970s and remains the mainstay for the prevention of APAP-induced hepatotoxicity. It is metabolised in the liver to the glutathione substrate cysteine. Glutathione is required for the detoxification of NAPQI to produce less toxic cysteine and mercapturic acid conjugates. POD patients in the UK receive an intravenous 21-h regimen of 150 mg/kg over 1 h, then 50 mg/kg over 4 h, then 100 mg/kg over 16 h (total dose 300 mg/kg) [5]. Although effective at preventing APAP-induced hepatotoxicity when used within 8 h of overdose, this regimen was largely empirical and not based on robust initial dose-ranging studies. The 21-h NAC regime is associated with the following challenges. i) Adverse drug reactions, since it is commonly associated with unpleasant and occasionally severe dose-related adverse drug reactions [6]. Nausea/vomiting occurs in more than half of recipients and anaphylactoid reactions in about a third. While cutaneous features are most common (flushing, itching), systemic effects occur in up to 5% of recipients. Severe reactions result in treatment interruption, treatment refusal, and extended hospital stays. ii) Medication error, since the regimen is complex, involving three separate weight-related infusions over different time frames. As a result, there is a high risk of medication error and deaths have been reported. iii) Prolonged duration, since the regime is time consuming, taking at least 21 h, leading to significant hospital bed occupancy (around 47,000 bed days per year in England) [7].

To address these shortcomings of the standard NAC regimen, a shorter 12-h intravenous regimen has been developed (the ‘SNAP’ regimen). In this 12-h NAC regimen, the initial loading dose (NAC 100 mg/kg in 200 mL) is given over 2 h, followed by a second dose (200 mg/kg in 1000 mL) infused over 10 h. This 12-h NAC regimen has been demonstrated to be effective in reducing the incidence of vomiting and anaphylactoid reactions compared with the standard intravenous acetylcysteine schedule [8]. The shorter duration of acetylcysteine infusion offers simpler administration, a probable reduction in administration errors, and potentially a substantial decrease in the length of hospital stay. Since September 2015, this 12-h NAC regimen has been delivered as standard clinical care at the Royal Infirmary of Edinburgh, UK.

Mangafodipir was originally developed as a magnetic resonance imaging (MRI) contrast agent and approved for that indication by the US Food and Drug Administration (FDA) and the European Medicines Agency (EMA). Approximately 240,000 patients have received single doses of mangafodipir with no significant safety concerns. Mangafodipir has been demonstrated to prevent APAP-induced liver injury in mice by acting as a superoxidase dismutase (SOD) mimetic. In mice, this protection is at a time point when NAC is no longer active, presumably corresponding with the oxidative phase of injury [9]. Therefore, mangafodipir may provide protection against POD in humans at a time when NAC treatment is less effective.

Calmangafodipir (Ca4Mn(DPDP)5) is a unique chemical species derived from mangafodipir, where 80% of the manganese in mangafodipir has been replaced with calcium. Based on the similarities between calmangafodipir and mangafodipir, it is anticipated that calmangafodipir would also exhibit SOD-dependent pharmacologic actions similar to those of mangafodipir [10]. Calmangafodipir (referred to in this protocol as PP100–01) has been studied in a phase 2 safety and efficacy study (PLIANT) of chemotherapy-induced peripheral neuropathy in patients with advanced metastatic colorectal cancer. In the PLIANT study, calmangafodipir was well tolerated across all three doses of 2, 5, and 10 μmol/kg [11]. Calmangafodipir, at a dose of 5 μmol/kg, prevented the development of chemotherapy-induced peripheral neuropathy without apparent influence on tumour outcomes.

To support clinical trials with calmangafodipir, this compound has undergone standard toxicity testing consisting of repeat dose studies of up to 6 months duration in rats and 9 months duration in dogs with intravenous dosing once every fortnight. Calmangafodipir showed good tolerability in both species with a no observed adverse effect level in the 6- and 9-month studies of 144- and 30-fold, respectively, above the highest clinical dose of 10 μmol/kg. Exposures obtained in terms of whole blood manganese levels as well as plasma Cmax and area under the curve (AUC) of the main metabolite PLED relative to the corresponding clinical exposures at 10 μmol/kg gave additional reassurance of the good tolerability of calmangafodipir.

When investigated in the Ames Salmonella assay and in a micronucleus assay in human lymphocytes, calmangafodipir showed no relevant genotoxic activity.

Investigations on fertility and early embryonic development in the rat with mangafodipir, a compound with similar circulating metabolites to that of calmangafodipir, revealed no negative effects up to the highest dose (100 μmol/kg) investigated [12]. However, in standard testing for embryofetal development toxicity, skeletal abnormalities have been observed in the rat at intravenous doses from 10 μmol/kg while in the rabbit incomplete ossification was seen after 20 μmol/kg [12]. These effects are related to manganese exposure since MnCl_2_ was shown in the rat to induce the same skeletal abnormalities as mangafodipir at equivalent molar doses. No adverse effects were observed when pregnant rats were administered mangafodipir up to day 7 of gestation, i.e. prior to major organogenesis taking place in this species.

The objective of the PP100–01 for Overdose of Paracetamol (POP) trial is to develop a therapeutic regimen for POD where a 12-h NAC infusion protocol is combined with PP100–01 to evaluate safety and tolerability and to provide information regarding the potential efficacy of this combination for preventing liver injury. The primary objective is to assess the adverse events (AEs) and serious adverse events (SAEs) associated with PP100–01 co-treatment with the 12-h NAC treatment regime in patients with POD. A secondary study objective is to determine if there is evidence of PP100–01 having efficacy by measurement of clinical and exploratory biomarkers. The exploratory biomarkers (microRNA-122 (miR-122), keratin-18 (K18), and glutamate dehydrogenase (GLDH)) are supported for use in the assessment of drug-induced liver injury in clinical trials, both by the EMA and the FDA. As they have been demonstrated to have enhanced sensitivity for identifying liver injury in the context of APAP overdose, their measurement may allow sub-clinical liver injury to be quantified [13].

## Methods

### Ethical and regulatory approval

**Fig. 1:**
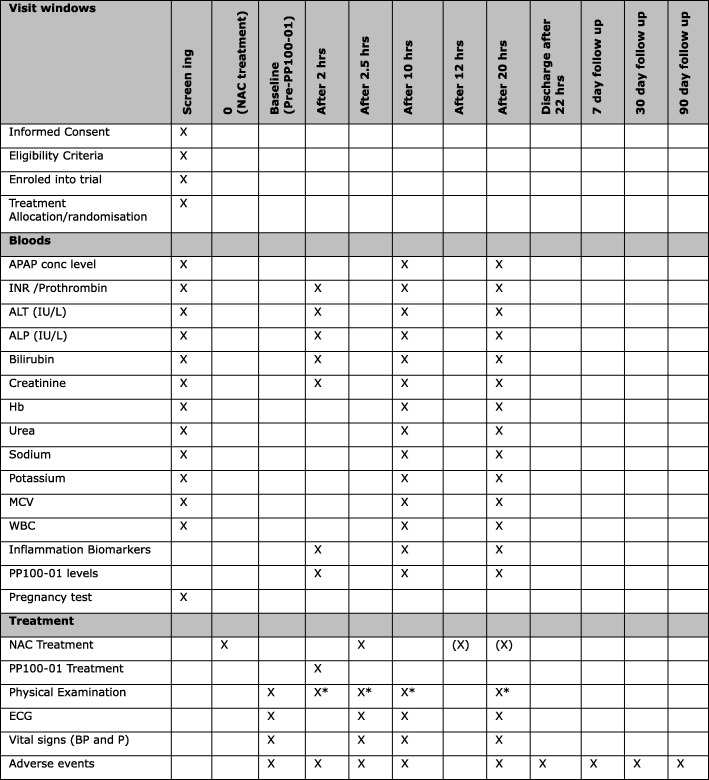
SPIRIT figure for the POP Trial. *If AEs/SAEs identified that require a full physical examination to be completed (X), NAC continued as per TOXBASE (12 h ± 30 min); 2- and 2.5-h assessments should be performed ± 15 min of the time point. PP100–01 treatment should be ± 10 min of the time point. 10- and 20-h assessments should be performed ± 30 min of the time point. ALP alkaline phosphatase, ALT alanine transaminase, APAP paracetamol/acetaminophen, BP blood pressure, ECG electrocardiogram, Hb haemoglobin, INR international normalised ratio, MCV mean cell volume, NAC N-acetylcysteine, P Pulse, WBC white blood cell

This study, EudraCT number 2017–000246-21 and ClinicalTrials.gov identifier NCT03177395, is funded by the Sponsor, PledPharma AB, Stockholm, Sweden, and was approved by the UK medicines regulator, Medicines and Healthcare products Regulatory Agency (MHRA; 25 April 2017), and West of Scotland Research Ethics Committee 1, Glasgow, UK (approval 11 April 2017). The trial was designed by collaborators at the Clinical Toxicology unit in the Royal Infirmary of Edinburgh and the Edinburgh Clinical Trials Unit. The protocol was designed in accordance with the Standard Protocol Items: Recommendations for Interventional Trials (SPIRIT) (Additional file 1). The trial overview is available as a SPIRIT figure (Fig. 1).

### Rationale for study drugs and doses

#### Acetylcysteine treatment

**Fig. 2:**
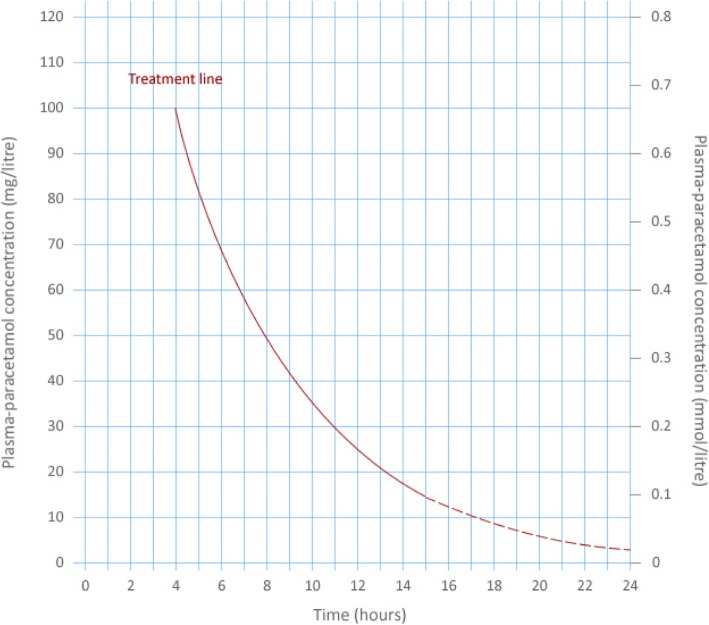
The paracetamol treatment nomogram used in the United Kingdom. After a single acute overdose, the patient’s plasma/serum paracetamol concentration is plotted on the graph using the time from overdose to blood draw. If above the treatment line the patient receives acetylcysteine treatment at a dose of 300 mg/kg body weight

After a single acute APAP overdose with a known time of drug ingestion, in the UK a nomogram is used to identify patients who require NAC treatment based on the plasma APAP concentration and time from ingestion (Fig. 2). Patients with timed plasma concentrations above the treatment line receive treatment with the full course of intravenous NAC. Those patients below the treatment line receive no NAC treatment and can be considered for medical discharge.

In patients presenting to hospital up to 8 h after a single overdose, the need for NAC treatment can be assessed once the plasma APAP concentration is known. In patients presenting later than 8 h (as well as staggered POD), NAC treatment is initiated if the patient reports ingesting a potentially toxic dose (150 mg/kg body weight). Once blood results are available NAC treatment can be stopped or continued.

In this clinical trial NAC is to be delivered by the 12-h SNAP regimen because this regimen is associated with few adverse reactions. This superior safety profile (compared to the 21-h regimen) will aid the identification of safety signals directly relating to PP100–01 and NAC co-treatment.

#### PP100–01 treatment

With regards to the choice of dose, in the phase II PLIANT trial in patients with advanced metastatic colorectal cancer, calmangafodipir (PledOx®; 2, 5, or 10 μmol/kg) was infused intravenously as a pre-treatment single dose over approximately 5 min. PledOx® was well tolerated across all three doses. There were no differences between PledOx®-treated arms and placebo in terms of AEs [11]. The mechanism of action of calmangafodipir in preventing chemotherapy-induced peripheral neuropathy is comparable to its proposed mechanism in APAP toxicity, namely acting as a SOD mimetic, which prevents cellular oxidative stress. Although the target organ is different, in this study the doses of PP100–01 will be the same as that in PLIANT.

### Study design

The study is an open-label, randomised, exploratory, rising dose design, NAC-controlled, phase 1 safety and tolerability study in patients treated with NAC for POD. Consent will be obtained prior to any study-specific procedures being carried out by delegated trained clinical staff and recorded on a consent form in triplicate, with one copy given to the participant, one placed in the Investigator Site File (ISF), and one placed in the hospital notes. Entry into the study is dependent on the patient’s blood results confirming the need for NAC. A total of 24 patients will be assigned into one of three dosing cohorts of eight patients (*n* = 6 for NAC plus PP100–01; *n* = 2 for NAC alone). Patients that have signed the informed consent form will be treated with NAC plus one of a rising series of doses of PP100–01. Each PP100–01 treatment dosing cohort will have eight patients, six randomised to NAC plus PP100–01 and two randomised to NAC alone. The doses of PP100–01 are 2, 5, and 10 μmol/kg.

### Identifying patients

Treating clinicians at the Emergency Department of the Royal Infirmary of Edinburgh will identify potentially eligible patients. A trained member of staff will then assess the patient for study eligibility.

Inclusion criteria:

1. Any patient with capacity admitted to hospital within 24 h of either a single acute POD or more than one dose of APAP (staggered) and deemed to require treatment with NAC.
2. Provision of written informed consent
3. Males and females of at least 16 years of age

Exclusion criteria:

1. Patients that do not have the capacity to consent to participate in the study
2. Patients detained under the Mental Health Act or deemed unfit by the Investigator to participate due to mental health
3. Patients with known permanent cognitive impairment
4. Patients who are pregnant or nursing
5. Patients who have previously participated in the study
6. Unreliable history of POD
7. Patients presenting more than 24 h after POD
8. Patients who take anticoagulants (e.g. warfarin) therapeutically or have taken an overdose of anticoagulants
9. Patients who, in the opinion of the responsible clinician/nurse, are unlikely to complete the full course of NAC, e.g. expressing wish to self-discharge
10. Prisoners
11. Non-English-speaking patients (study information material will only be produced in English in view of the known and stable demographic of the Edinburgh self-harm population)

#### Blood samples

As part of routine clinical care all patients presenting to hospital following a POD have the following blood tests on arrival to hospital or 4 h after overdose (whichever is the longer time).

- APAP concentration
- Urea, sodium, potassium, and creatinine
- Liver function tests (LFTs): alanine transaminase (ALT) activity, bilirubin, and alkaline phosphatase (ALP) activity.
- Clotting screen: international normalised ratio (INR) and prothrombin time (PT)
- Haemoglobin, white blood cells (WBC), and mean cell volume (MCV)

The clinical decision to treat a patient with NAC is largely based on their APAP concentration in combination with their ALT activity and INR.

**Fig. 3:**
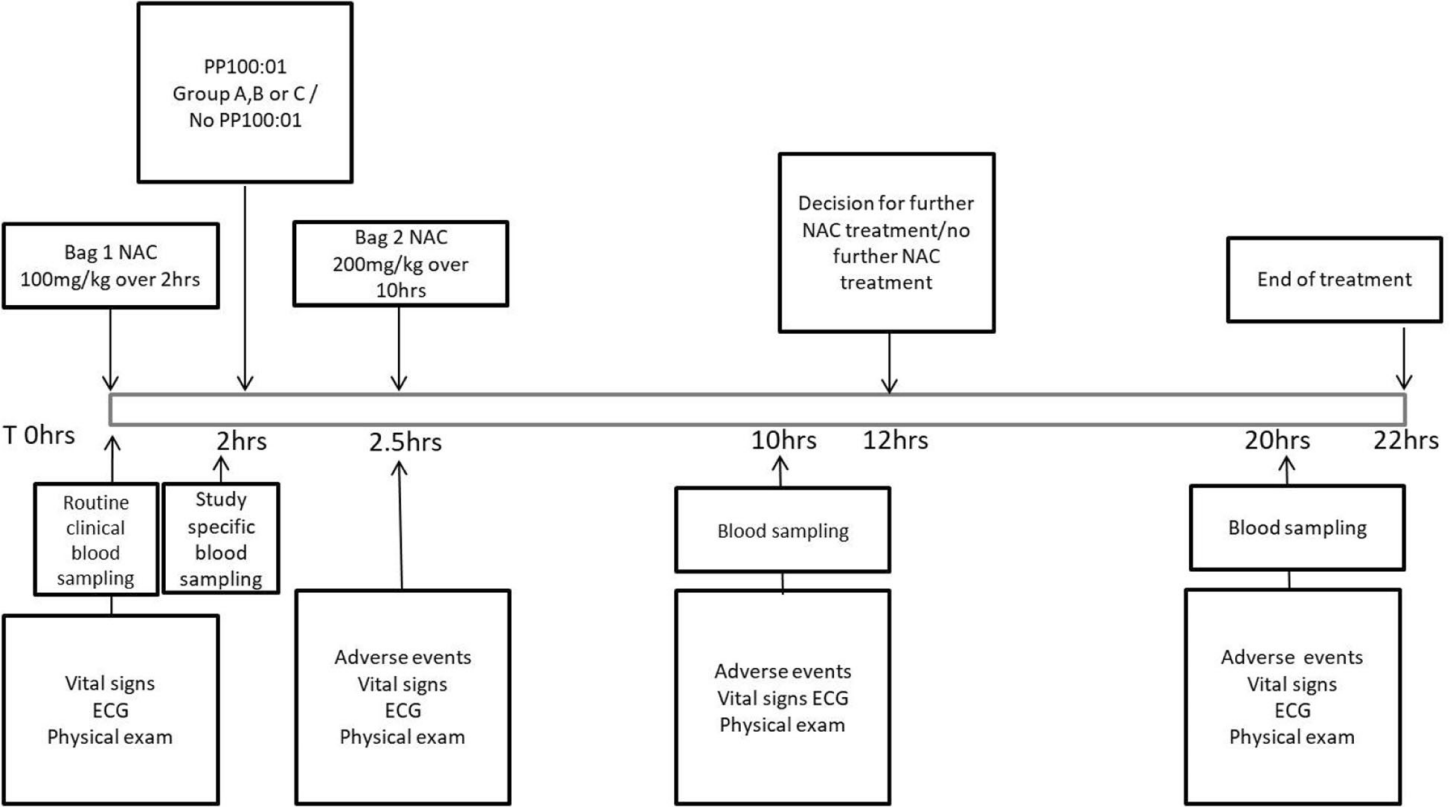
Diagram of the POP trial study design over a total of 22 h including blood sampling times and recording of adverse events. ECG electrocardiogram, NAC N-acetylcysteine

As part of routine clinical care, blood sampling will be taken to measure the above analytes at 10 h and 20 h after starting NAC (Fig. 3).

As part of a study examining exploratory biomarkers (miR-122, K18, and GLDH), a study-specific blood sample will be collected (10 mL split over serum and plasma) immediately prior to administering PP100–01. Similarly, at the 10-h and 20-h venepuncture episodes, study-specific blood samples will be collected.

### Baseline assessments

Study-specific vital signs will be assessed at baseline (between 1 and 2 h after starting NAC) and again at 2.5, 10, and 20 h after starting NAC (Fig. 3). The following vital signs will be evaluated in a supine position after at least 5 min of rest: blood pressure (systolic and diastolic; mmHg), heart rate (beats per minute), respiration rate (per minute), pulse oximetry (%), temperature (°C), and temperature route. Height (estimated) and weight (kg) are collected at baseline. A baseline physical examination will be completed assessing the cardiovascular, respiratory, and gastrointestinal systems. Any AEs and SAEs identified during the examination will be recorded/reported. Additional full physical examinations will be completed if AE/SAEs were reported, requiring a more thorough examination to be carried out. This will include assessment of local toxicity at the site of intravenous administration. All women of childbearing potential will be screened for pregnancy by urine or serum human chorionic gonadotropin (hCG) test as part of the eligibility assessment. An electrocardiogram (ECG) will be performed on resting patients at baseline, and at 2.5 h, 10 h, and 20 h, and any clinically significant findings will be recorded.

### Randomisation

The allocation sequence for each dosing cohort will be created using computer-generated random numbers, using blocking to ensure the required 6:2 ratio of NAC + PP100–01:NAC alone. The randomisation list is held centrally at the Edinburgh Clinical Trials Unit (ECTU) to conceal treatment allocations until these are implemented via the ECTU secure web-based randomisation system.

#### Treatment phase

Treatment starts with the first NAC bag of 100 mg/kg in 200 mL (“loading dose”) at time point ‘0’. After 2 h and immediately prior to PP100–01 (if randomised to this treatment), a study-specific blood sample will be collected. PP100–01 treatment will be administered intravenously as a bolus infusion over 5 min at the dose specified by the dosing cohort:

- Group A: PP100–01 (2 μmol/kg calmangafodipir) after the “loading” dose of NAC
- Group B: PP100–01 (5 μmol/kg calmangafodipir) after the “loading” dose of NAC
- Group C: PP100–01 (10 μmol/kg calmangafodipir) after the “loading” dose of NAC

If the patient is randomised to the NAC-alone group there is no intravenous injection 2 h after starting NAC. In all patients, the 12-h NAC regimen will be continued with the second dose: 200 mg/kg NAC diluted in 1000 mL delivered intravenously over 10 h. As per routine clinical practice, there is one blood sample taken 2 h before the end of the second NAC bag (the 10-h time point) and a second blood sample taken 10 h later (the 20-h time point) (Fig. 3).

### Extension of NAC treatment

At the end of the 12-h NAC regimen the decision to continue NAC will be made by assessment of the clinical blood sample taken at the 10-h time point. NAC will be continued at 200 mg/kg in 1000 mL intravenously over a further 10 h if any of the following criteria are reached:

- the ALT has more than doubled since the admission measurement, or
- the ALT is two times the upper limit of normal or more, or
- the INR is greater than 1.3
- APAP concentration > 20 mg/mL

### Adverse events

Adverse events/adverse reactions (AE/AR) are assessed at baseline, and at 2 h, 2.5 h, 10 h, 20 h, and 22 h, and as part of 7-, 30-, and 90-day follow-up. A full physical examination will be carried out if AEs or SAEs are identified and this will be recorded on the electronic case report form (e-CRF). All SAEs and suspected unexpected serious adverse reactions (SUSARs) will be reported for pharmacovigilance review within 24 h of identification. All events are followed up until resolution or until no longer medically indicated.

### Follow-up

Patients will be followed up using their electronic records. The following data will be collected 7, 30, and 90 days after randomisation: re-presentation to hospital (for any reason), re-presentation with liver injury, repeat overdose, death, and transfer to liver transplantation unit. Any AEs or SAEs identified will be recorded and reported.

### Primary objective

To determine the safety and tolerability of PP100–01 add-on treatment to the 12-h NAC treatment regimen in patients treated for POD. The primary outcome is any AEs or SAEs.

### Secondary objectives

Secondary outcomes include:

- Clinical observations (pulse rate, blood pressure, respiratory rate, pulse oximetry, temperature) and haematological and clinical biochemistry parameters.
- Exploratory biomarkers in serum/plasma (miR-122, K18, GLDH)
- Incidence of hepatotoxicity
- Duration of hospital stay (days)
- Re-presentation to hospital (for any reason), re-presentation with liver injury, repeat overdose, death, and transfer to liver transplantation unit, and AEs and SAES up to 90 days.

Exploratory secondary outcomes will be collected to improve the design of future clinical studies:

1. To determine the rate of occurrence of hepatotoxicity (defined by raised biochemical markers) in patients treated with PP100–01 and 12-h NAC administration regimens.
2. To compare the incidence of anaphylactoid reactions in the co-treatment and 12-h NAC regimens in APAP poisoned patients.
3. To determine the occurrence of hepatotoxicity as determined at the end of the 12-h NAC administration regimen.
4. To measure length of hospital stay in patients receiving co-treatment and 12-h NAC treatment regimens.
5. Proportion of patients with a 50% increase in ALT after 10 h post-treatment with NAC, compared with the admission value
6. Proportion of patients with ALT > 100 at 10 h post-treatment with NAC
7. Proportion of patients with INR > 1.3 at 10 h post-treatment with NAC
8. Proportion of patients with APAP concentration > 20 mg/mL

### Independent Safety Data Monitoring Committee (SDMC)

An independent SDMC will be appointed to review accumulating AE data and patient safety. The SDMC will evaluate the safety in relation to the PP100–01 dosing step increase. The committee will give recommendations on the continuation or termination of the study by detection of any safety signals as early as possible in accordance with the SDMC Charter. The proposed composition of the SDMC will be: independent hepatologist, independent statistician, independent clinical pharmacologist/toxicologist. During the period of recruitment into the study, interim analyses of in-hospital mortality/morbidity and any other information that is available on major outcome events (including SAEs believed to be due to treatment) will be supplied, in strict confidence, to the Chairperson of the SDMC, along with any other analyses that the committee may request.

All further patient enrolment will be paused pending advice from the SDMC if one of the following stopping rules have been met: 1) patient death, admission to a critical care unit or admission to a liver transplantation unit due to any reason; or 2) one SUSAR that definitely or probably relates to either PP100–01 or NAC or both.

All SMDC data reviews will be documented and all meetings will have written minutes, which will be filed in the trial master file (TMF) upon completion of the study.

### Statistical analyses

Patients will be included in the full analysis population, the primary population for analysis of efficacy, if they have received any PP100–01 or NAC. Data will be analysed according to the randomised treatment group. The stringent per-protocol population includes patients from the full analysis population for whom the study protocol has been followed without any major violations. The population for safety analysis will be patients who have received any PP100–01 or NAC. Data will be analysed according to the treatment received (NAC plus PP100–01, or NAC alone). Any patient who withdraws during the treatment phase of the study will be included in the safety population (AEs and laboratory parameters). Data for all patients will be listed, and a list of withdrawn patients, with all reasons for withdrawal, will be given. Data will also be listed for those patients who, after having consented to participate, underwent baseline examinations required for inclusion into the study but who, because a criterion for exclusion was met or for other reasons, were not included in the study.

With the clinical safety data that are available we do not expect any SAEs, but we note that PP100–01 has not been administered in POD patients treated with NAC. We deem that six patients per group in this initial dose escalation study will allow initial exploration of effects on biomarkers and potential dose-limiting toxicity.

A CONSORT diagram [14] depicting the flow of participants and representing the number of patients split by treatment group through the study will be reported. Descriptive statistics will be used to report baseline characteristics by treatment group and overall. Continuous variables will be summarised by the mean, standard deviation, median, and minimum and maximum; categorical variables will be summarised using the number and percentage in each category. Log transformation will be used where appropriate. Baseline characteristics will include participant details, overdose details, concomitant medications during the preceding 30 days, physical examination including cardiovascular, respiratory, and gastrointestinal systems, vital signs, ECG, and clinical bloods.

We will keep missing data to an absolute minimum, but where there are missing data those records will be removed from the analysis; if missing data rates are substantial the effect of this will be investigated using sensitivity analyses.

Binary outcomes (including the primary outcome) will be reported by treatment group and overall using the proportion and exact 95% confidence interval. Binary outcomes will be compared within dosing cohorts between the PP100–01 + NAC and the NAC-alone patients using a difference in proportions and its exact 95% confidence interval. Binary outcomes will be compared between each of the PP100–01 dosing groups (A, B, and C) and the combined NAC-alone group in the same way. Continuous outcomes will be reported by treatment group and overall using the mean and 95% confidence interval. Continuous outcomes will be compared within dosing cohort between the PP100–01 + NAC and the NAC-alone patients using the difference in means and its 95% confidence interval. Continuous outcomes will be compared between each of PP100–01 dose groups (A, B, and C) and the combined NAC-alone group in the same way. The continuous outcome analyses listed above will be repeated using the change from baseline in each continuous outcome.

### The primary outcome

The number and percentage of patients experiencing an AE will be summarised by treatment group and overall. The primary analysis will be to report AE and SAE rates by treatment group and overall using the method described above for binary outcomes. If the limited sample size or a small number of observed events leads to a lack of interpretability of the formal analysis confidence intervals, the analysis of the primary outcome will be restricted to descriptive summaries only.

The number and percentage of patients experiencing an AE will be summarised by subsets of AEs according to the following criteria:

- SAEs
- AEs starting after the commencement of NAC treatment and within 7 days of consent
- SAEs starting after the commencement of NAC treatment and within 7 days of consent
- Intensity (mild/moderate/severe)
- Treatment given in response to AE (yes/no)
- Outcome (recovered/improved/unchanged/deterioration/death)
- Relationship to NAC (unrelated/possibly related/probably related/definitely related)
- Relationship to PP100–01 (unrelated/possibly related/probably related/definitely related)
- Action taken with NAC (none/interrupted/stopped entirely)
- Action taken with PP100–01 (none/interrupted/stopped entirely)
- Unexpected with, and related to, NAC
- Unexpected with, and related to, PP100–01
- SUSAR, subcategorised into SUSAR to NAC, SUSAR to PP100–01, and SUSAR to NAC and PP100–01

### Secondary outcomes

#### Safety

Vital signs (systolic blood pressure, diastolic blood pressure, pulse, respiratory rate, temperature, oxygen saturation) and haematology and clinical biochemistry parameters (APAP level, PT, alkaline phosphatase, creatinine, urea, WBC, potassium, bilirubin, haemoglobin, MCV, sodium) will be summarised and analysed at their measurement time points. The ECG result (normal/abnormal not clinically significant/abnormal clinically significant) will be summarised by treatment group and overall at 2.5, 10, and 20 h. The ECG summary results at each of these time points will also be cross-tabulated against the baseline ECG result, by treatment group and overall. The proportion of anaphylactoid reactions recorded will be summarised by treatment group and overall.

#### Efficacy

Hepatotoxicity will be assessed using ALT and INR. Descriptive summaries will be provided by treatment group and overall at each measurement time point during the treatment period (baseline, 10 h, 20 h). Change from baseline to each of the 10- and 20-h time points will be summarised by treatment group and overall for ALT and INR. At each measurement time point during the treatment period (baseline, 10 h, 20 h) ALT and INR will be analysed by treatment group and overall using the mean and 95% confidence interval. They will be compared within each dosing cohort between the PP100–01 + NAC and the NAC-alone patients using the difference in means and its 95% confidence interval. The markers will be compared between each of the PP100–01 2 μmol/kg, 5 μmol/kg, and 10 μmol/kg groups and the combined NAC-alone group in the same way. We will measure exploratory biomarkers with analysis as per ALT.

## Discussion

APAP is the most common drug taken in overdose in the UK [15]. Hospitalisation due to APAP overdose accounted for approximately 80,000 bed days in the UK in 2005–2006. Current treatment involves use of NAC in patients deemed at risk of potential liver damage. This is given by intravenous infusion over a period of 21 h. This regimen was designed in the 1970s, with a large loading dose of the antidote administered followed by two decreasing concentrations. The initial infusion is associated with adverse reactions, in particular nausea, vomiting, and anaphylactoid reactions. The latter are particularly troublesome and occur in up to 15% of patients treated. Therapy is discontinued and there is often confusion as to whether therapy can be restarted in a timely manner. Although highly effective in preventing liver damage if given sufficiently early, further disadvantages of the conventional intravenous NAC infusion regimen are the high rates of adverse reactions and the complexity of dosing and resulting high risk of medication errors.

The objective of this study is to develop a therapeutic regimen for POD where a NAC 12-h regime is combined with a SOD mimetic, PP100–01, to evaluate if reduction of the oxidative stress in the liver will be safe and well tolerated. It would also allow preliminary data to be collected on a new approach of giving NAC together with PP100–01 using the 12-h regimen, which included a slower initial intravenous infusion over 2 h instead of 1 h in the 21-h NAC regime.

The primary study outcome is therefore chosen to inform about the safety and tolerability of PP100–01 co-treatment with the NAC regime in patients with POD. In addition, data on the incidence of adverse effects caused by the NAC plus PP100–01 regimen, and changes in liver toxicity markers induced by APAP-induced liver injury within this modified NAC treatment will be obtained.

If the combination of PP100–01 and NAC is tolerated, then the next trial should focus on establishing a dose of PP100–01 that has efficacy. To achieve this aim, sensitive biomarkers of acute liver injury may be used as the outcome measures. The current trial will provide valuable data regarding the variability of these biomarkers in this population and may even demonstrate a ‘signal’ consistent with efficacy of PP100–01 for the prevention of APAP-induced liver injury.

In conclusion, this study is the first attempt to combine a superoxide dismutase (SOD) mimetic, (P100–01), co-treatment with a 12-h NAC regimen in patients with POD. Developing and refining therapeutic regimens in POD is important as the current 21-h regimen leads to adverse reactions and may not be therapeutically optimal or timely for lessening liver injury as a result. We anticipate this study will provide clinical trial evidence for the safety and tolerability of PP100–01 when combined with NAC treatment.

### Trial status

This paper describes study protocol version V3 (28 June 2017). The trial opened on 5 June 2017. The first patient was recruited on 8 June 2017. The expected end of recruitment May 2018.

## Additional file

Additional file 1: SPIRIT 2013 checklist: recommended items to address in a clinical trial protocol and related documents. (DOC 121 kb)

## Abbreviations

AE: Adverse event
ALP: Alkaline phosphatase
ALT: Alanine transaminase
APAP: Paracetamol/acetaminophen
ECTU: Edinburgh Clinical Trials Unit
GLDH: Glutamate dehydrogenase
GSH: Glutathione
INR: International normalised ratio
K18: Keratin-18
MHRA: Medicines and Healthcare products Regulatory Agency
miR-122: MicroRNA-122
NAC: N-acetylcysteine
NAPQI: N-acetyl-p-benzoquinoneimine
POD: Paracetamol overdose
PT: Prothrombin time
SAE: Serious adverse event
SDMC: Safety Data Monitoring Committee
SNAP: Scottish and Newcastle Anti-emetic Pre-treatment for Paracetamol Poisoning
SOD: Superoxide dismutase
SUSAR: Suspected unexpected serious adverse reaction
